# Palbociclib Plus Letrozole for the Treatment of Metastatic Breast Cancer: An Illustrative Case Scenario

**DOI:** 10.6004/jadpro.2016.7.5.7

**Published:** 2016-07-01

**Authors:** Kristi Orbaugh,1, Joanne C. Ryan,2, Lynn Pfeuffer,3

**Affiliations:** 1Community Hospital Oncology Physicians, Zionsville, Indiana; 2Pfizer Inc, New York, New York; 3Allegheny Health Network Cancer Institute, Pittsburgh, Pennsylvania

## Abstract

**CASE STUDY**

Betty, a 66-year-old white female, was diagnosed with stage IIB, T2N1M0, estrogen receptor/progesterone receptor–positive, HER2-negative breast cancer in 2007. It was detected on screening mammography when she was age 59, and she was confirmed to be postmenopausal at the time. She has no family history of breast cancer. Betty has never smoked but enjoys drinking alcohol, normally with dinner and typically limited to 1 drink of hard liquor per day. Her medical history includes type 2 diabetes mellitus and hypertension, which are adequately controlled with metformin and lisinopril.

Betty is married and a retired teacher. She has four healthy adult children and five grandchildren. She noted that although she was always thin as a child, she was never able to lose the weight she gained during her pregnancies. Currently, her body mass index (BMI) is around 29 kg/m2. She enjoys ballroom dancing with her husband, gardening, and walking her dog and she is an active member at her church. Betty and her family were shocked to hear about her diagnosis. After a discussion with her oncologist, a treatment plan was devised. Her Eastern Cooperative Oncology Group performance status at diagnosis was 0.

Initial treatment consisted of neoadjuvant chemotherapy with dose-dense doxorubicin/cyclophosphamide (AC) × 4 cycles followed by weekly paclitaxel × 12 cycles. Betty tolerated treatment relatively well. However, she was hospitalized once after cycle 3 of AC for neutropenic fever. Her subsequent cycle was followed with pegfilgrastim. Repeat imaging after AC treatment revealed a good overall response. Other adverse effects from treatment included fatigue and nausea for a few days after each cycle. Residual grade 1 neuropathy secondary to her treatment with paclitaxel, with a potential contribution from her history of diabetes, was a long-term complication.

Following completion of her neoadjuvant therapy, she had a lumpectomy and then radiation therapy. Adjuvant endocrine therapy with the aromatase inhibitor (AI) anastrozole was given for 5 years, which she completed in late 2012. Bone health was monitored with dual-energy x-ray absorptiometry screening. Mild osteopenia was noted during AI therapy, and she was given twice-daily calcium plus vitamin D supplementation. Annual surveillance diagnostic breast mammography along with biannual history and physical examinations showed no signs of disease recurrence.

In 2007, Betty was one of 26% of women who were diagnosed with breast cancer in the United States, at a time when breast cancer accounted for 15% of cancer deaths (American Cancer Society [ACS], 2007). Similarly, in 2016, estimates predict that 29% of new cancer diagnoses in women are expected to be breast cancer, with 14% of cancer deaths being attributed to breast cancer (ACS, 2016). Excluding nonmelanoma skin cancers, breast cancer ranks as the most common cancer diagnosis in women and the second leading cause of cancer-related death, surpassed only by lung cancer (ACS, 2016).

Betty represents a fairly typical patient with breast cancer: a postmenopausal woman with hormone receptor (HR)–positive, HER2-negative disease. Approximately 80% of breast cancers are classified as estrogen receptor (ER)-positive, and most are also progesterone receptor (PR)-positive (Dunnwald, Rossing, & Li, 2007). Moreover, the likelihood of ER/PR-positive breast cancer increases with advancing age. Although the *HER2* oncogene emerged during the late 1990s as a novel target, approximately 75% of patients have HER2-negative breast cancer and therefore are not suitable for anti-HER2 therapy with agents such as trastuzumab (Herceptin; Slamon et al., 2001).

Despite lacking a family history of breast cancer and maintaining a relatively active lifestyle, Betty had several risk factors for breast cancer: weight gain as an adult (overweight body mass index [BMI] category), alcohol consumption, and history of type 2 diabetes (ACS, 2015). For Betty, there is a low probability of inherited breast cancer susceptibility attributable to the *BRCA1* or *BRCA2* mutation, in light of her unremarkable family history and age at diagnosis. Overall, only about 5% to 10% of breast cancer cases are believed to stem from these breast cancer susceptibility mutations (ACS, 2015).

## MULTIMODALITY THERAPY

Whether the year was 2007 or 2015, Betty’s treatment course and clinical experience are fairly typical for a patient diagnosed with locally advanced breast cancer. Multimodality therapy—surgery, radiation therapy, and systemic therapies (chemotherapy, hormonal therapy, biologics)—is the standard course of action for patients with nonmetastatic breast cancer (National Comprehensive Cancer Network [NCCN], 2015). Per the most current NCCN Clinical Practice Guidelines, dose-dense AC followed by weekly paclitaxel is among the preferred neoadjuvant regimens for HER2-negative disease, with the administration of a taxane following AC demonstrated to reduce the risk of relapse compared with AC alone (Henderson et al., 2003; Mamounas et al., 2005). In the North American Breast Cancer Intergroup Trial E1199, weekly paclitaxel was shown to improve both disease-free and overall survival compared with standard paclitaxel every 3 weeks when given after AC for T1–3, N1–2, M0, or high-risk node-negative disease (Sparano et al., 2008).

Betty’s hospitalization due to the development of febrile neutropenia during AC was not surprising. Given the propensity for AC to cause neutropenia, current NCCN recommendations are to administer growth factor support with all four of the AC cycles (NCCN, 2015). It is well appreciated that Betty’s peripheral neuropathy is a common toxicity of paclitaxel (Bristol-Myers Squibb, 2011). In the E1199 trial, the incidence of grade ≥ 2 neuropathy was 27% with weekly paclitaxel, whereas grade 3/4 neuropathy occurred at an incidence of 8%; these rates were higher than those seen with paclitaxel every 3 weeks or with docetaxel every 3 weeks or weekly (Sparano et al., 2008).

Initiation of adjuvant therapy, specifically a 5-year course of AI therapy, was also consistent with standard practice for a postmenopausal patient. Although a 5-year course of adjuvant tamoxifen is the only established regimen for adjuvant endocrine therapy in premenopausal women, there are a number of different established options for postmenopausal women, which include using an AI from the onset or after several years of tamoxifen (NCCN, 2015). Importantly, deeming Betty postmenopausal required not only a history of amenorrhea for at least 12 months but also follicle-stimulating hormone and estradiol levels in the postmenopausal range (NCCN, 2015).

## METASTATIC DISEASE

**Case Study Continued: Pain Affects Lifestyle**

Betty was doing well until the spring of 2015, when she began to experience persistent lower back pain. A lumbar magnetic resonance image (MRI) revealed bone metastasis to her L4/L5 region. A positron emission tomography (PET)−computed tomography (CT) scan also showed a metastatic lesion to the left humerus. There were no impending fractures. Her CA 15-3 level, a biomarker highly associated with breast cancer, was elevated at 47 U/mL. Her pain was managed with hydrocodone/acetaminophen at 7.5 mg/325 mg every 4 to 6 hours as needed. Unfortunately, her active lifestyle was affected by the pain. Her Eastern Cooperative Oncology Group (ECOG) performance status was 1 when she discussed treatment plans with her oncology care team.

**Novel Targeted Therapies**

Unlike ER-negative tumors, for which there is an early peak in the recurrence rate followed by a gradual decline, ER-positive breast tumors are known to carry a steady risk for recurrence of about 2% per year over prolonged follow-up extending beyond 10 years (Cadoo, Fornier, & Morris, 2013). In cases of distant recurrence, ER-positive tumors also carry a higher propensity for bone metastases relative to ER-negative tumors (Cadoo et al., 2013). With no curative treatment options available for patients with metastatic breast cancer, the primary goals of treatment are to extend survival while maintaining or improving quality of life (NCCN, 2015). For patients with HR-positive recurrent or metastatic breast cancer, the use of single-agent or combination chemotherapy is typically reserved for use in the endocrine-refractory disease setting.

However, new options have recently become available for treating patients with HR-positive breast cancer—supplementing endocrine therapy with either the mammalian target of rapamycin (mTOR) inhibitor everolimus (Afinitor) or the reversible cyclin-dependent kinase (CDK) 4/6 inhibitor palbociclib (Ibrance). Both everolimus and palbociclib are oral targeted agents that have been shown to prolong progression-free survival (PFS) when added to endocrine therapy in randomized phase II studies for advanced breast cancer (Baselga et al., 2012; Finn et al., 2015). Use of everolimus in conjunction with endocrine therapy was designed to target resistance mechanisms and therefore prolong the response duration for the endocrine agent (Baselga et al., 2012). Conversely, palbociclib acts against the cell-cycle dysregulation that is inherent to cancer by restoring cellular senescence or permanent growth arrest (Anders et al., 2011; Thangavel et al., 2011).

**Figure 1:**
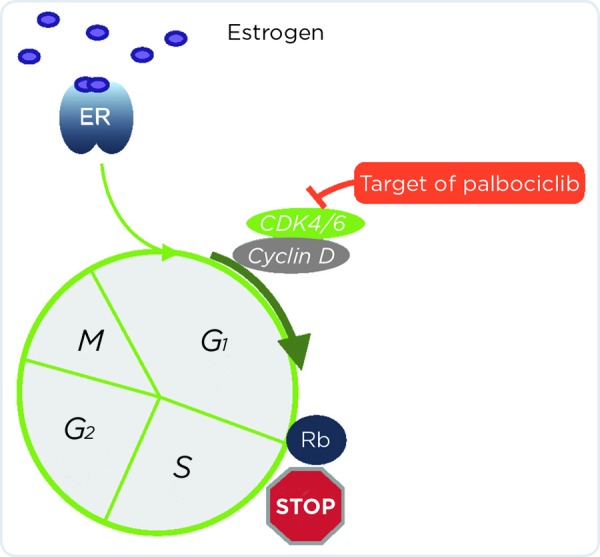
The cell cycle and regulatory process. There are four phases in the cell cycle, that serve as checkpoints to ensure that cells are ready to move to the next phase: the S phase (DNA synthesis), M phase (mitosis), and the G1 and G2 gap phases (Cadoo et al., 2014). Positive and negative regulators tightly control the process of cell division and the cell cycle. Cyclin-dependent kinases (CDKs) are positive regulators of cell cycling, with CDK4/6 playing a regulatory role in the G1 to S transition, which is also influenced by a negative regulator, Rb (retinoblastoma protein). Various cellular signaling pathways result in upregulation and activation of cyclin D, which complexes with CDK4/6 (the targets of palbociclib), facilitating the phosphorylation of Rb proteins and inactivating them; this process allows progression through the cell cycle (Anders et al., 2011). By inhibiting CDK4/6, palbociclib has been shown to inhibit Rb phosphorylation and block progression of the cell from the G1 to the S phase of the cell cycle (Pfizer Inc., 2016). ER = estrogen receptor.

When cells divide, there are four phases: the S phase (DNA synthesis); M phase (mitosis); and the G1 and G2 gap phases, which serve as checkpoints to ensure that cells are ready to move to the next phase (Cadoo, Gucalp, & Traina, 2014). Cyclin-dependent kinases are positive regulators of cell cycling, with CDK1 acting at the G2 to M transition. Cyclin-dependent kinases 2, 4, and 6 regulate the G1 to S transition, which is also influenced by a negative regulator: the retinoblastoma (Rb) tumor suppressor gene. Cyclin-dependent kinases 4/6 are capable of disabling Rb proteins through phosphorylation and as a result allowing progression through the cell cycle (Anders et al., 2011). By blocking CDK4/6, palbociclib has been shown to inhibit Rb phosphorylation when combined with letrozole in ER-positive breast cancer xenografts, with the combination reducing Rb phosphorylation, and resultant downstream signaling and tumor growth, to a greater extent than either agent alone (Figure 1; Pfizer Inc., 2016). Preclinical studies have demonstrated that palbociclib has preferential effects against ER-positive cell lines in vitro (Finn et al., 2009).

*Everolimus*: The BOLERO-2 trial, which was a randomized phase III trial, evaluated the combination of everolimus and exemestane in 724 postmenopausal women with ER-positive, HER2-negative advanced breast cancer considered refractory to anastrozole or letrozole. Refractory disease was defined as recurrent disease during or within 12 months of adjuvant use or within 1 month after completing treatment for advanced disease (Baselga et al., 2012). Based upon the results from this trial of a median PFS of 6.9 months with everolimus plus exemestane and 2.8 months with placebo plus exemestane, the NCCN recommended consideration of everolimus in patients meeting the criteria applied in BOLERO-2. However, Betty is not considered to be refractory to endocrine therapy based on the definition in the BOLERO-2 trial. As a result, she would not be an appropriate candidate for everolimus.

*Palbociclib*: Betty’s medical team also considered palbociclib as a treatment option. Palbociclib was evaluated as a combination treatment with letrozole in the PALOMA-1/TRIO-18 randomized phase II trial (Finn et al., 2015). In this trial, 165 postmenopausal women with previously untreated ER-positive, HER2-negative advanced breast cancer received palbociclib plus letrozole or letrozole alone until disease progression, toxicity, withdrawal, or death (Finn et al., 2015). Women in both arms received letrozole at 2.5 mg once daily, with the combination arm also receiving palbociclib at 125 mg once daily. Letrozole was given continuously, whereas palbociclib was given daily for 3 weeks followed by a 1-week break, constituting a 28-day treatment cycle.

The final analysis demonstrated that the primary endpoint of median PFS was nearly doubled in the palbociclib plus letrozole arm (20.2 months vs. 10.2 months), translating into a significant 51% reduction in the risk of disease progression or death during follow-up relative to letrozole alone (*p* = .0004). Key efficacy data, summarized in Table 1, show significant improvement with the combination therapy in many of the secondary outcomes. The safety profile of palbociclib plus letrozole was described as predictable and manageable, producing predominantly hematologic adverse events (AEs). The most common AEs (≥ 10%) are summarized in Table 2.

**Table 1:**
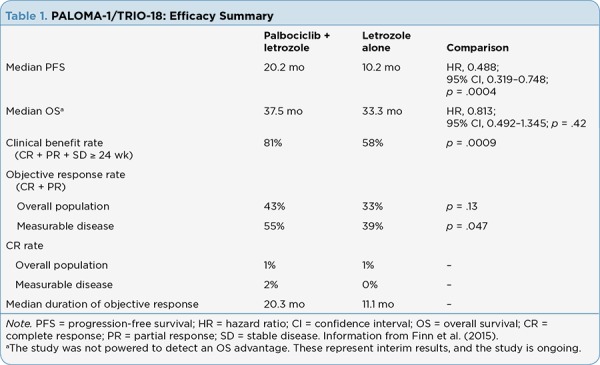
PALOMA-1/TRIO-18: Efficacy Summary

**Table 2:**
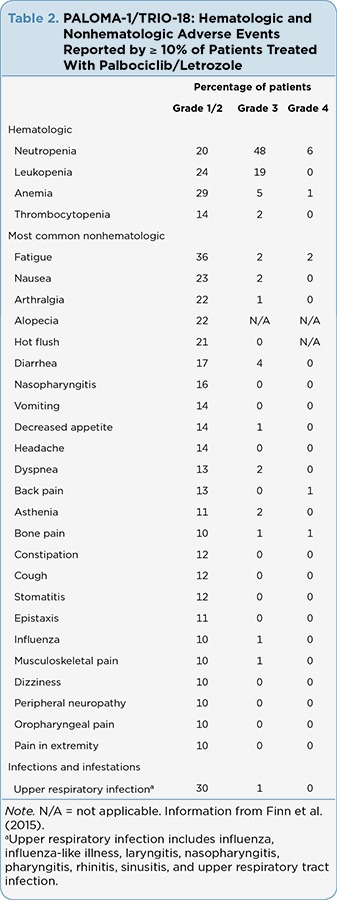
PALOMA-1/TRIO-18: Hematologic and Nonhematologic Adverse Events Reported by ≥ 10% of Patients Treated With Palbociclib/Letrozole

As palbociclib is primarily metabolized by the CYP3A pathway and the sulfotransferase enzyme SULT2A1, it is critical to review the patient’s list of reported medications and to inquire about diet and the use of herbal supplements (Pfizer Inc., 2016). Patients need to avoid grapefruit/grapefruit juice and St. John’s wort due to interactions with the metabolizing pathways (Table 3; Pfizer Inc., 2016). Furthermore, when using palbociclib, antibacterial and antiviral medications that are strong inhibitors of CYP3A should be avoided, or the palbociclib dose should be reduced if these interacting medications are needed. Additionally, palbociclib is a weak time-dependent inhibitor of CYP3A, and sensitive CYP3A substrates with a narrow therapeutic index, such as fentanyl, may need to be dose reduced because palbociclib may increase the exposure of CYP3A substrates.

**Table 3:**
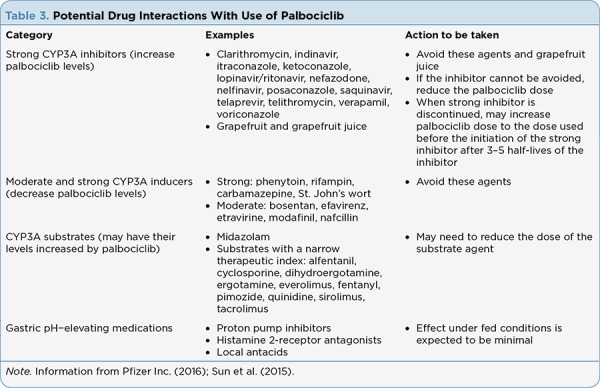
Potential Drug Interactions With Use of Palbociclib

**Case Study Continued: Candidate for Targeted Agents**

Betty’s medical team believed she would be an excellent candidate for treatment with palbociclib and letrozole. Her medical team also recommended intravenous zoledronic acid for bone health. Betty and her husband agreed with this treatment option. The oncology advanced practitioner (AP) met with Betty and her husband to provide information about the proposed regimen.

Treatment was palbociclib at 125 mg by mouth daily for 3 weeks on (days 1−21) and 1 week off (days 22−28) along with letrozole at 2.5 mg by mouth daily continuously. She was instructed to take the medications with food at the same time each day and not to chew or crush the capsules. Before the start of therapy and on day 1 of each cycle, Betty was advised to undergo laboratory evaluation with a complete blood cell count (CBC) and differential and also to have a CBC and differential on day 14 during the first 2 cycles. Her medical care team explained that this was to assess for neutropenia and any need for patient-specific precautions or dose adjustment.

Betty’s medication list was reviewed by her AP and the clinical pharmacist. Betty reported to her provider that she enjoys drinking 2 to 3 glasses of grapefruit juice a few times a week. It was explained that palbociclib is primarily metabolized via the CYP3A pathway in the liver, and grapefruit/grapefruit juice interferes with this pathway, which can thereby affect the level of palbociclib. As a result, Betty was instructed to stop drinking grapefruit juice and eating grapefruit while on treatment.

Betty was also advised against drinking alcohol because of her use of pain medications and risk for hematologic adverse effects. Betty’s provider and pharmacist also recommended that she switch to oxycodone (5 mg) every 4 hours as needed for her pain, because the acetaminophen component of hydrocodone/acetaminophen can mask febrile neutropenia. Betty was instructed to avoid acetaminophen and nonsteroidal anti-inflammatory medications. Betty’s care team also discussed her residual grade 1 sensory peripheral neuropathy (not affecting activities of daily living) that emerged during prior neoadjuvant chemotherapy.

## ROLE OF ADVANCED PRACTITIONERS

The provider-patient interaction at the onset of cancer treatment and the ongoing relationship are very important in the era of oral regimens. Adherence is vital to the success and benefit of any oral medication regimen. Although oral formulations have long been available for endocrine agents, oral targeted therapy is relatively new. APs are in the position to offer not only education but also ongoing monitoring to maximize the likelihood of adherence and patient satisfaction with the treatment regimen. For example, use of a pillbox and monthly calendar would be of particular value for someone like Betty, who will be self-administering two different drugs: one to be given daily and the other to be given with a 1-week break built into the treatment schedule.

The AP should discuss Betty’s prior experience with adjuvant endocrine therapy to gain insight into any potential existing barriers to adherence. Any history of poor adherence, whether to medication, screenings, or attendance at appointments, should be viewed as a red flag (Miaskowski, Shockney, & Chlebowski, 2008). It is important to discuss patients’ expectations of treatment that may affect their commitment to treatment and address any issues regarding their support system, underlying psychological problems, or the cost of medication. To enhance open and timely patient-provider communication, APs should ensure that patients have their up-to-date office contact information. Additionally, if Betty has not already received one, a list of organizations; support groups; or other relevant local, regional, or national resources would be worthwhile.

## ADVERSE EVENTS

Providing oral and written information on the treatment and potential AEs, including their management, is another strategy to improve patient adherence (Miaskowski et al., 2008). Although palbociclib has a novel mechanism of action distinct from that of classic cytotoxic chemotherapy agents, its influence on the cell cycle carries a propensity for influencing rapidly dividing cells, which include hematologic indices. In the PALOMA-1/TRIO-18 trial, the most common AE in patients treated with palbociclib plus letrozole was neutropenia (~75% all grades).

**Side Effects in Clinical Trials**

The mechanism of neutropenia associated with palbociclib is through cell-cycle arrest without apoptosis, which differs from the mechanism of neutropenia associated with chemotherapies that result in cell-cycle arrest, apoptosis, DNA damage, and minimal senescence (Hu, Sung, Jessen, & Sacaan, 2015). Most cases of neutropenia in the trial were of grade 3/4 severity, with a grade 1/2 incidence of 20% and grade 3 and 4 incidences of 48% and 6%, respectively (Finn et al., 2015). Conversely, neutropenia in the letrozole monotherapy arm was limited to a 4% grade 1/2 incidence and a 1% grade 3 incidence (Finn et al., 2015). Importantly, there were no reports of febrile neutropenia in the phase II trial (Finn et al., 2015), although febrile neutropenia has been reported in the palbociclib clinical program (Cristofanilli et al., 2016).

The second most common AE was leukopenia, with grade 1/2 leukopenia occurring in 24% of palbociclib patients and grade 3 leukopenia occurring in 19%, compared with 3% and 0%, respectively, in the letrozole group (Finn et al., 2015). Anemia was also common (29% grade 1/2, 5% grade 3, and 1% grade 4), and thrombocytopenia was common to a lesser extent (14% grade 1/2 and 2% grade 3) in the palbociclib plus letrozole group (Finn et al., 2015). In the letrozole-only group, grade 1/2 and grade 3 anemia occurred in 5% and 1% of patients, respectively, and grade 1/2 thrombocytopenia occurred in 1% of patients (Finn et al., 2015).

Nonhematologic toxicity was typically of grade 1/2 severity, as shown in Table 2 (Finn et al., 2015). Those with an all-grade incidence > 20% were fatigue, nausea, arthralgia, alopecia, diarrhea, and hot flush (Finn et al., 2015). For patients who develop persistent nausea or other gastrointestinal complaints, antiemetic medications as needed, diet education, avoidance of foods that could potentially cause gastrointestinal irritation, and weight monitoring are recommended.

The most common grade 3/4 nonhematologic AEs were fatigue (2% grade 3; 2% grade 4) and diarrhea (4% grade 3; 0% grade 4). Serious AEs reported in at least one patient in the palbociclib plus letrozole group were pulmonary embolism (4%; three patients), back pain (2%; two patients), and diarrhea (2%; two patients; Finn et al., 2015). Adverse events that led to treatment discontinuation in patients in the palbociclib plus letrozole group included neutropenia (6%), asthenia (1%), and fatigue (1%; Pfizer Inc., 2016). Of note, the safety profile of palbociclib plus letrozole in PALOMA-1/TRIO-18 was notably similar to that of palbociclib plus fulvestrant (Faslodex) in the subsequently conducted PALOMA-3 trial (Turner et al., 2015).

## PATIENT EDUCATION AND MONITORING

A patient like Betty may already be familiar with the types of AEs that can occur during treatment with palbociclib, given that several overlap with those expected during traditional systemic chemotherapy or endocrine therapy. However, as part of the process of patient education, APs should mention that palbociclib is one of various new agents that are distinct from chemotherapy, and as such, potential AEs may be different.

Adverse events can generally be managed by dose interruptions, delays, reductions, and discontinuations. Betty might expect to receive prophylactic growth factor support and may even specifically ask for it, given her experiences with adjuvant chemotherapy. The prophylactic use of granulocyte colony-stimulating factors was not permitted in the PALOMA-1/TRIO-18 clinical trial, but they were approved for use to manage treatment-emergent neutropenia. It should be thoroughly explained to patients that although the risk of neutropenia is relatively high, patients in the clinical trial did not report any episodes of febrile neutropenia (Finn et al., 2015).

The AP should explain to patients such as Betty that they will be closely monitored for neutropenia and other AEs during therapy, with a CBC at the start of each cycle and a day 14 CBC during the first two cycles as part of the routine care recommended in the approved product labeling (Pfizer Inc., 2016). As palbociclib is distributed via a system of specialty pharmacies, it is critical that patients be instructed to call the office and notify their providers once the drug has been received to allow for scheduling of the day 14 CBC. After the first two cycles, CBCs should be performed on day 1 and on an as-needed basis. As a precaution, patients should be educated to report early signs of infection and other hematologic complications, such as easy bruising and bleeding, which may occur with thrombocytopenia.

Before the start of therapy, the AP noted that Betty had residual grade 1 peripheral neuropathy from her adjuvant chemotherapy. In PALOMA-1/TRIO-18, the incidence of peripheral neuropathy was 10% with palbociclib/letrozole vs. 5% with letrozole monotherapy; however, all cases were of grade 1/2 severity (Finn et al., 2015). Consequently, it is important for providers to obtain an accurate neurologic assessment at baseline before starting Betty’s new therapy and on day 1 of each cycle.

**Case Study Continued: Importance of Patient Monitoring**

Betty reveals in her discussion with the AP that she uses a weekly pillbox and calendar to help her remember when to take her pills. Printed educational materials and a calendar were reviewed in detail, including the importance of the break week for palbociclib. Betty’s first cycle with palbociclib and letrozole was well tolerated. Her day 14 CBC revealed that she had grade 1 anemia (hemoglobin 9.8 g/dL) and that her neutrophils decreased to 1,250/mm³ (grade 2). These results were reviewed with her provider, and per the dosing guidelines, her dose of palbociclib remained the same. Betty offered no complaints and continued with therapy.

**Figure 2:**
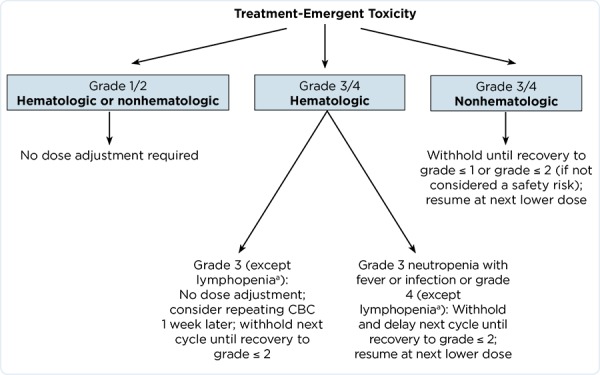
Dose modification for toxicity. CBC = complete blood cell count. Information from Pfizer Inc. (2016). aUnless associated with clinical events, e.g., opportunistic infections.

Betty should be informed that AE management includes dose reductions, delays, reductions, or discontinuations. Based on the recommendations in the product label, dose reduction or interruption is not warranted for patients developing grade 1 or 2 hematologic changes (Figure 2; Pfizer Inc., 2016).

At the beginning of cycle 2, Betty’s hemoglobin normalized, her neutrophil count improved to grade 1 (1,480/mm³), and there was no delay to the start of the second cycle. The CBC drawn on day 14 indicated that Betty again had grade 1 anemia and grade 3 neutropenia, with an absolute neutrophil count (ANC) of 950/mm³. Betty had no signs or symptoms of infection and continued on her current dose with a repeat CBC on day 21.

The repeat CBC indicated continued grade 3 neutropenia, with an ANC of 920/mm³. No dose adjustments were required (day 21 begins the scheduled break in palbociclib dosing). Betty was advised to continue to monitor for signs and symptoms of infection. Betty’s CBC on day 1 of cycle 3 indicated improvement in her ANC to grade 2 at 1,200/mm³, and the start of cycle 3 was not delayed.

**Focus on Neutropenia**

In the absence of fever or infection, palbociclib may be continued in the setting of grade 3 neutropenia without dose interruption or reduction, but consideration should be given to repeating the CBC after 1 week; if it is still grade 3, delay the start of the next cycle until it improves to grade ≤ 2 (Figure 2; Pfizer Inc., 2016).

In PALOMA-1/TRIO-18, the median onset to any-grade neutropenia was 15 days (range: 13–117 days), and the median duration of grade ≥ 3 neutropenia was 7 days. In the setting of grade 3 neutropenia (ANC < 1,000−500/mm³) complicated by fever (≥ 38.5° C) and/or infection, palbociclib should be held, and treatment should be delayed until recovery to grade ≤ 2. In addition, palbociclib should be restarted at 100 mg/day for the first reduction and 75 mg/day for the second reduction. As no further dose reductions are possible, palbociclib should be discontinued as needed.

**Case Study Continued: Cycle 3 and Beyond**

During cycles 3 and 4, Betty experienced grade 2 neutropenia without fever or signs or symptoms of infection. Betty’s neuropathy was unchanged and stable from baseline (grade 1). At each cycle, the AP confirmed that Betty understood and was compliant with dosing instructions. After cycle 4, Betty underwent rescanning, which demonstrated an improvement in her bone lesions by approximately 30%. In addition, Betty is now only taking two to three pain pills daily, and her husband reports that she is able to take her dog for longer walks again. Betty continues on treatment with palbociclib and letrozole and is currently receiving cycle 5 of this combination therapy.

## DISCUSSION

This case illustrates several benefits of the use of palbociclib and oral targeted agents for the treatment of metastatic breast cancer. Although metastatic breast cancer is incurable, it is possible to achieve disease control and associated symptomatic benefits in this patient population. The provider-patient relationship is of heightened importance with this type of therapy, to ensure open communication regarding dosing, monitoring, and AE assessment and reporting.

One favorable aspect of the palbociclib plus letrozole regimen is its convenience, with each agent to be taken only once daily at the same time. As the 1-week treatment break for palbociclib may be confusing for some patients, the drug is supplied in bottles of 21 capsules, thus reinforcing the 21-day dosing. Additionally, frequent education regarding the break and recommendation of tools such as a pillbox or calendar can further assist with patient adherence. For patients who have received prior therapy, engaging in a discussion of these past experiences offers an opportunity to explain any similarities or differences in terms of treatment expectations and to reemphasize that palbociclib is a unique targeted nonchemotherapeutic agent.

Because most AEs observed with palbociclib in PALOMA-1/TRIO-18 were of grade 1/2 severity, dose interruptions and reductions are not always required. Sufficient time should be allotted to provide patient education specific to letrozole, even for patients who have received endocrine therapy in the past, and to discuss issues pertaining to support; the logistics of filling the prescription; and strategies to mitigate cost concerns, including introducing the patient to support programs to assist with the cost of prescriptions.

In February 2016, a new US Food and Drug Administration approval was granted for palbociclib for the treatment of HR-positive, HER2-negative advanced or metastatic breast cancer in combination with fulvestrant in women with disease progression following endocrine therapy (Pfizer Inc., 2016). This indication supports the use of palbociclib in a broader range of women, such as those who are pre- and perimenopausal and is based on the results of the phase III PALOMA-3 trial (Cristofanilli et al., 2016; Turner et al., 2015).

As of this writing, a placebo-controlled phase III trial of palbociclib/letrozole and three other phase III trials of palbociclib in the treatment of breast cancer are underway and will provide further insight into its efficacy and safety profile, including its potential use with other endocrine agents and in early breast cancer. Current ongoing trials of palbociclib include patients with previously untreated locally recurrent or metastatic breast cancer, patients with stage II or III early breast cancer, and patients with residual (in breast or nodes) invasive breast cancer after neoadjuvant chemotherapy.

**Acknowledgments and Disclosures**

This article was based upon studies sponsored by Pfizer Inc. Editorial support was provided by Laurie Orloski, PharmD, and Simon Slater, PhD, of Complete Healthcare Communications, LLC, and was funded by Pfizer Inc. Dr. Ryan is an employee of and owns stock in Pfizer Inc. Ms. Pfeuffer is a consultant for Pfizer Inc. Ms. Orbaugh is on the speakers bureaus for Pfizer, Teva, Bristol-Myers Squibb, and Novartis.
